# Preserving inferior right hepatic vein enabled bisegmentectomy 7 and 8 without venous congestion: a case report

**DOI:** 10.1186/s40792-021-01184-w

**Published:** 2021-04-21

**Authors:** Masayoshi Terayama, Kyoji Ito, Nobuyuki Takemura, Fuyuki Inagaki, Fuminori Mihara, Norihiro Kokudo

**Affiliations:** Department of Surgery, National Center for Global Health and Medicine, Hepato-Biliary-Pancreatic Surgery Division, 1-21-1 Toyama, Shinjuku-ku, Tokyo, 162-8655 Japan

**Keywords:** Hepatectomy, Inferior right hepatic vein, Hepatocellular carcinoma, Three-dimensional simulation, Intraoperative ultrasonography, Dye injection technique, Counter staining

## Abstract

**Background:**

In hepatectomy, the preservation of portal perfusion and venous drainage in the remnant liver is important for securing postoperative hepatic function. Right hepatectomy is generally indicated when a hepatic tumor involves the right hepatic vein (RHV). However, if a sizable inferior RHV (IRHV) exists, hepatectomy with preservation of the IRHV territory may be another option. In this case, we verified the clinical feasibility of anatomical bisegmentectomy 7 and 8 with RHV ligation, averting the right hepatic parenchyma from venous congestion, utilizing the presence of the IRHV.

**Case presentation:**

A 70-year-old man was presented with a large hepatic tumor infiltrating the RHV on computed tomography during a medical checkup. The patient was diagnosed with hepatocellular carcinoma (HCC), T2N0M0, stage III. Right hepatectomy was first considered, but multi-detector computed tomography (MDCT) also revealed a large IRHV draining almost all of segments 5 and 6, suggesting that IRHV-preserving liver resection may be another option. The calculated future remnant liver volumes were 382 mL (26.1% of the total volume) after right hepatectomy and 755 mL (51.7% of the total volume) after anatomical bisegmentectomy 7 and 8; therefore, we scheduled IRHV-preserving anatomical bisegmentectomy 7 and 8 considering the prevention of postoperative liver failure and increased chance of performing repeat resections in cases of recurrence. Preoperative three-dimensional simulation using MDCT clearly revealed the portal perfusion area and venous drainage territories by the RHV and IRHV. There was an issue with invisibility of the anatomical resection line of segments 7 and 8, which was completely dissolved by intraoperative ultrasonography using Sonazoid and the portal dye injection technique with counter staining. The postoperative course in the patient was uneventful, without recurrence of HCC, for 30 months after hepatectomy.

**Conclusions:**

IRHV-preserving anatomical bisegmentectomy 7 and 8 is a safe and feasible procedure utilizing the three-dimensional simulation of the portal perfusion area and venous drainage territories and the portal dye injection technique.

**Supplementary Information:**

The online version contains supplementary material available at 10.1186/s40792-021-01184-w.

## Background

Hepatectomy is currently the most effective curative treatment for hepatocellular carcinoma (HCC) [1–3]. Recently, parenchyma-sparing hepatectomy has been established with great advantages in terms of the prevention of postoperative liver failure and increased opportunity to perform repeat resections in cases of recurrence [4, 5]. The functional preservation of the remnant liver requires hepatic venous flow, as well as arterial and portal flow because of the lack of venous perfusion-induced liver dysfunction and delayed hepatic regeneration in the congested area [6–8].

The inferior right hepatic vein (IRHV) drains the right posterior–inferior area of the liver [9–11]. When a liver tumor infiltrates the right hepatic vein (RHV), right hepatectomy is usually needed, because RHV resection causes congestion of the entire right side of the liver. If a sizable IRHV exists in such cases, the RHV may be ligated and divided while preserving the draining territory of the IRHV, usually segments 5 and 6 defined by Couinaud’s nomenclature [12]. However, IRHV-preserving hepatectomy is technically demanding in terms of preoperative evaluation of the portal perfusion and venous drainage in the preserved parenchyma and the intraoperative method to determine the liver transection line [13, 14].

Herein, we report a case of IRHV-preserving anatomical bisegmentectomy 7 and 8 in a patient with HCC who required combined RHV resection. Preoperative three-dimensional (3D) simulation was effective in precisely visualizing the portal perfusion area and venous drainage territories by the RHV and IRHV [15]. Intraoperative ultrasonography (IOUS) using Sonazoid and the portal dye injection technique with counter staining enabled us to identify the liver transection line to selectively resect segments 7 and 8.

## Case presentation

**Fig. 1:**
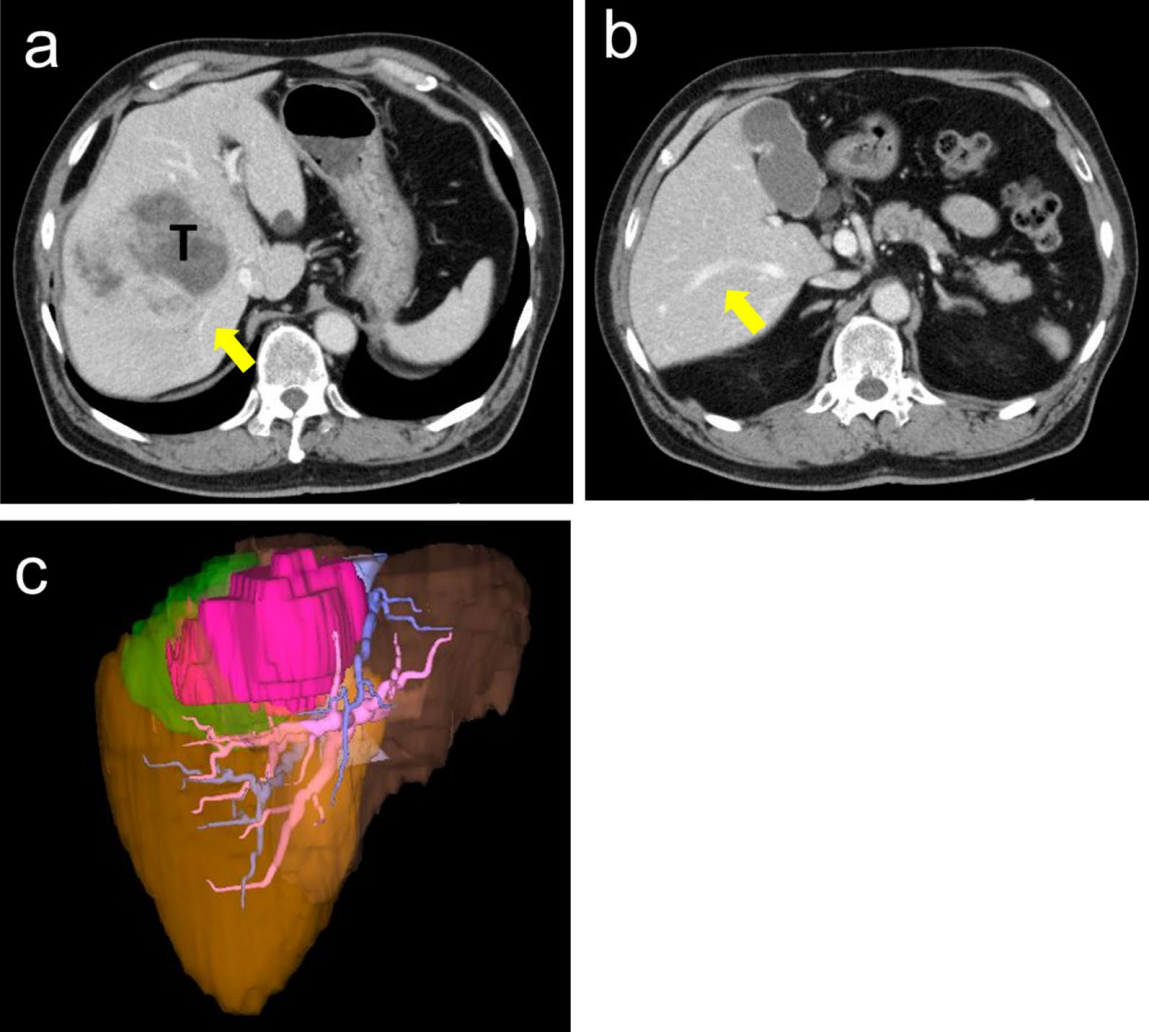
Hepatocellular carcinoma occupying segments 7 and 8 involved the RHV. **a** Computed tomography showed the tumor located in segments 7 and 8. The tumor involved the RHV (yellow allow). **b** The thick IRHV (yellow arrow) was observed. **c** 3D CT imaging of the RHV (green), IRHV (orange) drainage areas, and tumor (pink). RHV; right hepatic vein. IRHV; inferior right hepatic vein. 3D CT, three-dimensional computed tomography

The patient was a 70-year-old man who was presented with a large hepatic nodule on computed tomography (CT) during a medical checkup. He was referred to our hospital for further examination in October 2017. He had neither clinical symptoms nor a past medical history. Laboratory tests showed normal levels of albumin (4.5 g/dL), total bilirubin (0.6 mg/dL), indocyanine green retention at 15 min (7.1%), and alpha-fetoprotein (6.3 ng/mL) and an elevation in protein induced by vitamin K absence-II (3517 mAU/mL). Contrast-enhanced multi-detector CT (MDCT) and magnetic resonance imaging confirmed the presence of a large hepatic tumor, 10 cm in diameter, with arterial enhancement and washout, which was consistent with the characteristics of HCC (Fig. 1a). There were no ascites, extrahepatic invasion, intrahepatic metastasis, or remote metastasis. He was diagnosed with HCC, T2N0M0, stage III, but the etiology was unclear.

**Fig. 2:**
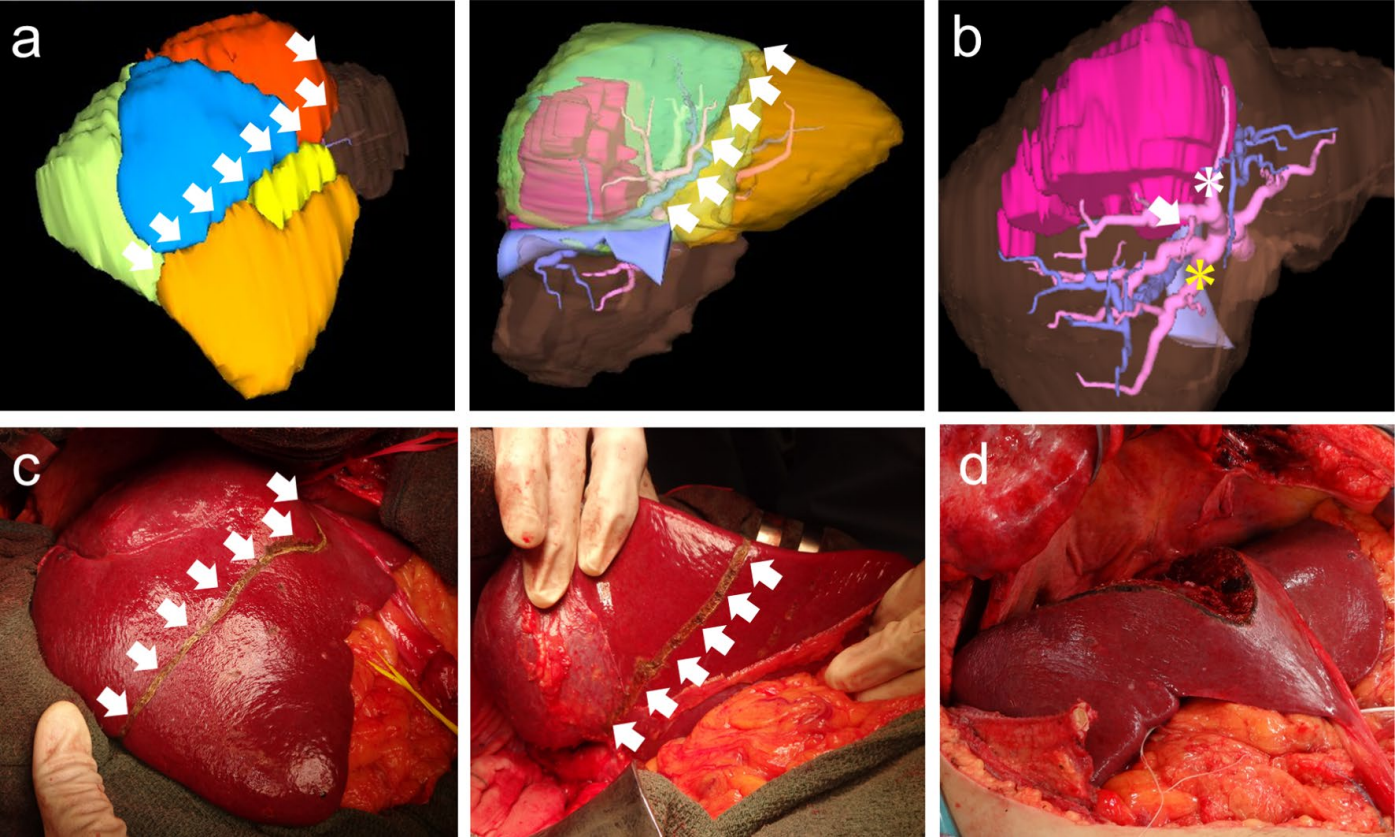
Determination of the transection line and liver resection. **a** (Left) 3D CT imaging of segment 5 (yellow), segment 6 (orange), segment 7 (green), the dorsal portion of segment 8 (blue), and the ventral portion of segment 8 (red). (Right) 3D CT imaging on the backside. The white arrows show the planned resection line. **b** 3D CT imaging of P5 (white arrow), dorsal P8 (white asterisk), and P6 (yellow asterisk). **c** The resection line was marked according to the demarcation line after portal staining. Left, frontside. Right, backside. **d** The resection surface of the remnant liver. 3D CT, three-dimensional computed tomography

He was classified with Child–Pugh grade A disease, and right hepatectomy was first considered; however, MDCT also revealed a large IRHV draining segment 6 directly into the inferior vena cava (Fig. 1b). Preoperative simulation using 3D CT confirmed that almost all of segments 5 and 6 were drained by the IRHV (Fig. 1c), suggesting the possibility of decongestion of the segments after division of the RHV [15]. The calculated future remnant liver volumes were 382 mL (26.1% of the total volume) after right hepatectomy and 755 mL (51.7% of the total volume) after anatomical bisegmentectomy 7 and 8. Right hepatectomy was considered to be over-resection for the patient, and we scheduled anatomical bisegmentectomy 7 and 8 (Fig. 2a).

The operation was performed with a J-shaped incision with left thoracotomy. Mobilization of the right side of the liver was carefully performed to prevent IRHV injury. The RHV and IRHV were then isolated and looped. IOUS revealed a tumor compressing the middle hepatic vein (MHV) without any apparent invasion. Although the tumor was attached to the portal branches of segments 7 and 8 (P7 and P8), the roots of the portal veins were intact. We then attempted staining of the territories of segments 7 and 8 using indigo carmine to determine the liver transection line [16]. P5 branched from the ventral branch of P8; therefore, the ventral and dorsal P8 were separately stained to demarcate between segments 5 and 8 (Fig. 2b). The approach to P7 was difficult due to its anatomically deep position; therefore, P6 was punctured as counter staining to reveal the border between segments 6 and 7 [17]. The pre-marked liver transection line was consistent with the boundary simulated in the preoperative 3D imaging (Fig. 2c, Additional file 1: Figure S1a).

After occlusion of the hepatoduodenal ligament through the intermittent Pringle maneuver, anatomical bisegmentectomy 7 and 8 was conducted using the crush clamping method. First, liver transection was conducted along the Rex–Cantlie line, and the MHV was identified. The MHV was intact from tumor invasion and was successfully preserved. Glisson’s pedicles of segment 7 were then exposed and divided. For segment 8, the dorsal and ventral branches were separately divided to preserve the branch for segment 5. Finally, the RHV was divided and closed with a running suture, and the specimen was removed. The resection surface of the liver is shown in Fig. 2d. The resected specimen showed the complete resection of the tumor (Additional file 1: Figure S1b). The pathological findings were consistent with those of HCC without lymphatic or venous invasion. The background liver was not cirrhotic. The postoperative course was uneventful, and the patient was discharged on postoperative day 16. The patient is alive with no recurrence of HCC 30 months after hepatectomy.

## Discussion

Anatomical bisegmentectomy 7 and 8 is a rare procedure, because it requires ligature of the RHV, which impairs the venous drainage of segments 5 and 6. Therefore, right hepatectomy is generally performed when tumors are located across segments 7 and 8. In the present case, we verified the clinical feasibility of anatomical bisegmentectomy 7 and 8 with RHV resection, preserving the caudal region of the right lobe (segments 5 and 6) and utilizing the presence of the IRHV.

The IRHV is clinically important as a draining hepatic vein other than the left hepatic vein, MHV, and RHV, especially in cases of hepatectomy requiring division of the RHV [12]. A study involving angiographic evaluation showed that the IRHV chiefly drains segment 6, and parts of segments 7 and 5 are also within the territory of the IRHV [10, 18]. Without the presence of the IRHV, anatomical bisegmentectomy 7 and 8 with ligation of the RHV leads to congestion and subsequent atrophy in segments 5 and 6, which may cause hepatocyte injury [19]. Sano et al. reported that hepatic congestion impairs tissue saturation with oxygen [8]. Hwang et al. suggested that the impairment of hepatic drainage prevents postoperative regeneration of the remnant liver [6]. Thus, liver resection with division of the RHV often requires right hepatectomy. In our case, right hepatectomy was not indicated, because the calculated future remnant volume was too small (26.1% of the total volume), which was a risk factor of postoperative morbidity and mortality [20–23]. In addition, preservation of the hepatic parenchyma as much as possible is important to conduct repeated resections considering the high rate of recurrence within 5 years after hepatectomy [24]. Percutaneous transhepatic portal vein embolization (PTPE) is reported to be useful to induce hypertrophy of future remnant volume (FLR). Yamashita et al. stated PTPE added around 10% FLR volume to HCC patients [25]. However, in the presented case, the speculated volume of post-PTPE FLR was less than 40% of the total liver volume, which was insufficient to perform right hepatectomy [26]. In this case, the patient had a well-developed IRHV, and preoperative 3D simulation using MDCT revealed that the IRHV drained almost all of segments 5 and 6. Therefore, anatomical bisegmentectomy 7 and 8 with RHV resection was successfully performed. Without the IRHV, our patient required hepatectomy with vascular reconstruction using synthetic artificial grafts or autologous vascular grafts to resect the tumor, although these have disadvantages such as infection, long-term stricture, and thrombosis [27, 28].

However, the clinical application of IRHV-preserving anatomical bisegmentectomy 7 and 8 is challenging and technically demanding because of the absence of anatomical landmarks between the upper two segments (S7, 8) and lower two segments (S5, 6) in the right liver. Additionally, the deep location of the vascular pedicles of segments 7 and 8 made it difficult to perform selective clamping of the pedicles before liver transection due to the risk of bleeding and bile duct injury [29, 30]. To identify the anatomical resection line that is not demarcated by the anatomical landmarks in the outer liver appearance, a dye injection method involving puncturing the portal branches guided by IOUS, was proposed by Makuuchi et al. [16]. There were several reports on a technique to determine the liver transection line in IRHV-preserving hepatectomy. Nakayama et al. decided the transection line based on the discolored congested area made by cramping the RHV and right hepatic artery [31]. Sugimachi et al. also demonstrated the modified hanging maneuver with taping of the IRHV to determine the transection line between segment 6 and 7 [32]. These techniques use the venous perfusion areas as a landmark for the transection line to completely resect the venous congested areas. However, the recent report, proposed by Kawaguchi et al., showed that blood inflow was partially preserved in venous congested areas, suggesting the possibility of preserved liver function [33]. Dye injection technique focused on Glissonian branches as a landmark for the transection line, and this approach has an advantage in terms of preserving future remnant liver and minimizing the exposure of major Glissonian branches on the transection plane. In the present case, the boundary between segments 5 and 8 was identified through puncture of the ventral and dorsal branches of P8, because P5 branched from the ventral branch of P8. In addition, puncture of P7 was difficult due to its anatomically deep position, and therefore, we punctured P6 as counter staining to reveal the border between segments 6 and 7 [17]. Utilizing the dye injection technique, we successfully conducted IRHV-preserving anatomical bisegmentectomy 7 and 8, which is superior to non-anatomical resection in terms of better long-term survival and prevention of intrahepatic metastasis (34, 35).

## Conclusion

IRHV-preserving anatomical bisegmentectomy 7 and 8 is a safe and feasible procedure utilizing the three-dimensional simulation of the portal perfusion area and venous drainage territories and the portal dye injection technique.

## Supplementary Information

**Additional file 1: Figure S1.** (a) The imaging picture of dye staining. As we stained with indigo-carmine which was disappeared in a short time, the dye staining was almost washed out, while we repeated the puncture of the portal branch and the marking of the transection line by electrocautery. The imaging area of segment 8 (green) and segment 6 (blue) dye staining is shown. (b) The photograph of the tumor in the resected specimen (arrow).

## Data Availability

The datasets analyzed in the current study are not publicly available, because they contain information that may compromise the privacy of the patient but are available from the corresponding author on reasonable request.

## Abbreviations

3D: Three-dimensional
CT: Computed tomography
HCC: Hepatocellular carcinoma
IOUS: Intraoperative ultrasonography
IRHV: Inferior right hepatic vein
MHV: Middle hepatic vein
MDCT: Multi-detector computed tomography
RHV: Right hepatic vein
