# Supplementary figures and images for: Preserving inferior right hepatic vein enabled bisegmentectomy 7 and 8 without venous congestion: a case report
Source: Surg Case Rep. 2021 Apr 21;7:101. doi: 10.1186/s40792-021-01184-w (PMC8060379; doi:10.1186/s40792-021-01184-w)

# Supplement Figure 1

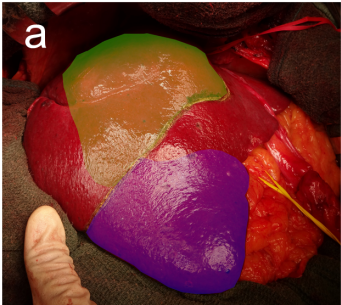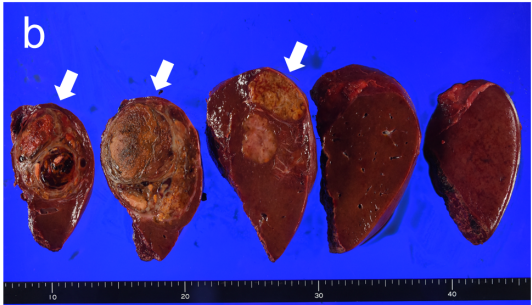

Supplement: Supplementary file 1 — Additional file 1: Figure S1. (a) The imaging picture of dye staining. As we stained with indigo-carmine which was disappeared in a short time, the dye staining was almost washed out, while we repeated the puncture of the portal branch and the marking of the transection line by electrocautery. The imaging area of segment 8 (green) and segment 6 (blue) dye staining is shown. (b) The photograph of the tumor in the resected specimen (arrow). [file 40792_2021_1184_MOESM1_ESM.pdf]
